# Widening the offer of human papillomavirus self‐sampling to all women eligible for cervical screening: Make haste slowly

**DOI:** 10.1002/ijc.34358

**Published:** 2022-11-23

**Authors:** Matejka Rebolj, Alexandra Sargent, Sisse Helle Njor, Kate Cuschieri

**Affiliations:** ^1^ Cancer Prevention Group, School of Cancer & Pharmaceutical Sciences, Faculty of Life Sciences & Medicine King's College London London UK; ^2^ Cytology Department, Manchester Royal Infirmary Manchester University NHS Foundation Trust Manchester UK; ^3^ University Research Clinic for Cancer Screening, Department of Public Health Programmes Randers Regional Hospital Randers Denmark; ^4^ Department of Clinical Medicine Aarhus University Aarhus Denmark; ^5^ Scottish HPV Reference Laboratory, Royal Infirmary of Edinburgh, NHS Lothian Scotland Edinburgh UK

**Keywords:** cervical cancer, cervical intraepithelial neoplasia, human papillomavirus, mass screening, self‐collection

## Abstract

Self‐collection of samples for human papillomavirus (HPV) testing has the potential to increase the uptake of cervical screening among underscreened women and will likely form a crucial part of the WHO's strategy to eliminate cervical cancer by 2030. In high‐income countries with long‐standing, organised cervical screening programmes, self‐collection is increasingly becoming available as a routine offer for women regardless of their screening histories, including under‐ and well‐screened women. For these contexts, a validated microsimulation model determined that adding self‐collection to clinician collection is likely to be cost‐effective on the condition that it meets specific thresholds relating to (1) uptake and (2) sensitivity for the detection of high‐grade cervical intraepithelial neoplasia (CIN2+). We used these thresholds to review the ‘early‐adopter’ programme‐level evidence with a mind to determine how well and how consistently they were being met. The available evidence suggested some risk to overall programme performance in the situation where low uptake among underscreened women was accompanied by a high rate of substituting clinician sampling with self‐collection among well‐screened women. Risk was further compounded in a situation where the slightly reduced sensitivity of self‐sampling vs clinician sampling for the detection of CIN2+ was accompanied with lack of adherence to a follow‐up triage test that required a clinician sample. To support real‐world programmes on their pathways toward implementation and to avoid HPV self‐collection being introduced as a screening measure in good faith but with counterproductive consequences, we conclude by identifying a range of mitigations and areas worthy of research prioritisation.

**Abbreviations**
CI: confidence interval
CIN: cervical intraepithelial neoplasia
HPV: human papillomavirus

## 1 THE FUTURE OF CERVICAL SCREENING RELIES ON SELF‐SAMPLING

The increased popularity of self‐sampling for cervical screening^1^ relies on decades of research that attest to the credibility of high‐risk human papillomavirus (HPV) testing for the detection of high‐grade cervical intraepithelial neoplasia (CIN) and cervical cancer.^2^ Traditionally, a trained clinician would take a cervical sample through intimate, speculum examination, which some women find physically and/or psychologically challenging.^3^ For reasons such as privacy and avoidance of embarrassment many women prefer self‐collection.^4^ While screening uptake has been declining in several countries,^5^, ^6^ the finding that some women who do not engage in ‘regular’ screening may be willing to provide a self‐collected sample^7^ is significant. Consequently, self‐sampling has earned support from health care providers, patient organisations and policy makers,^8^, ^9^, ^10^, ^11^ and is a critical component of the WHO's global strategy to eliminate cervical cancer.^12^

## 2 ROLE FOR HPV SELF‐SAMPLING IN ORGANISED SCREENING PROGRAMMES IN HIGH‐INCOME COUNTRIES

Here, we reflect on the evidence to support the implementation of self‐sampling in high‐income settings with free‐of‐charge, quality‐assured, population‐based screening programmes based on clinician sampling, which are saving thousands of lives annually.^13^ We discuss this evidence through the lens of a study by Rozemeijer et al who explored the implications of implementing HPV self‐sampling within the Dutch‐organised screening programme.^14^ The study employed the well‐validated MISCAN microsimulation model^15^ and established, under a wide range of scenarios, that cervical screening combining both collection methods could save more lives at a societally acceptable cost than screening using clinician collection alone.^14^

This said, the study also identified conditions under which self‐sampling introduced into a high‐quality screening programme may inadvertently become counterproductive.^14^ These conditions were defined as co‐existence of the following outcomes (Table 1):

1. a low uptake of self‐sampling among underscreened women, especially those with a higher‐than‐average background risk of cervical cancer, and
2. a substantial proportion of women who usually undergo clinician‐based screening switch to self‐collection, and
3. a lower detection of CIN2+ compared to clinician sampling.

**TABLE 1:** Outcomes of HPV self‐sampling which, if observed concurrently, could lead to an overall loss of effectiveness in a quality‐assured cervical screening programme achieving high coverage rates with clinician sampling

*Changes associated with self‐collection for HPV testing*	*Screening programme will be less effective if this is*
Screening participation among high‐risk, underscreened women	Low
Proportion of previously well‐screened women who would switch to self‐collection	High
Detection of CIN2+ compared with clinician sampling	Low

Abbreviations: CIN, cervical intraepithelial neoplasia; HPV, human papillomavirus.

Rozemeijer et al identified several combinations of critical thresholds for these three outcomes (Table 2).^14^ If all three thresholds were met concurrently, a screening programme offering self‐collection would save fewer quality‐adjusted life years, that is, be less effective in decreasing the population burden of cervical cancer than a programme relying solely on clinician collection. The exact values of these critical thresholds would likely be different had their study been undertaken more recently or in a different country. Nevertheless, they describe a consistent pattern in which even relatively modest levels of switching from clinician collection to self‐collection could become counter‐productive in good faith if, concurrently, self‐sampling were unable to motivate a significant proportion of underscreened, high‐risk women to participate and struggled to achieve a comparable detection of CIN2+. An independent microsimulation study from Norway by Burger and colleagues largely corroborated these conclusions.^16^

**TABLE 2:** Selected quantified thresholds for the uptake of HPV self‐sampling among well‐screened women provided by Rozemeijer et al^14^ with specific combinations of the relative sensitivity for the detection of CIN2+ and the uptake among underscreened women, beyond which a screening programme offering self‐collection would not remain effective, that is, would generate fewer quality‐adjusted life years than a programme without self‐collection

Relative sensitivity for the detection of CIN2+^a^	0.95‐0.96			0.89‐0.90		
Increased uptake among nonparticipants^b^	6% (including high‐risk women)^c^ ^,^ ^d^	3% (including high‐risk women),^c^ ^,^ ^d^ or 6% (only average‐risk women)	6% (no high‐risk women)	6% (including high‐risk women)^c^ ^,^ ^d^	3% (including high‐risk women),^c^ ^,^ ^d^ or 6% (only average‐risk women)	6% (no high‐risk women)
Switching among women who would otherwise undergo clinician collection^e^	[Always effective]^f^	>50%	>30%	>70%	>30%‐40%	>20%

Abbreviations: CIN, cervical intraepithelial neoplasia.

^a^ For self‐collection vs clinician collection. The authors did not report the outcomes of scenarios in which the relative detection of CIN2+ achieved with self‐sampling was lower than 0.89 compared with clinician collection. In all scenarios, the authors assumed that 92% of women with positive self‐collected HPV tests undergo clinician‐collection for triage cytology at baseline, and that 68% attend at early recall compared to 92% of the women who were screened with clinician collection at baseline.

^b^ As a proportion of the programme's target population.

^c^ The authors did not report the outcomes of scenarios in which the extra uptake among nonattending women was higher than 6% (this choice was in line with an earlier Dutch clinical study), or those where the extra uptake was lower than 3%.

^d^ When their scenarios included high‐risk women, the authors assumed that 29% of nonattenders who provide a self‐collected sample never attend regular screening and have a higher background risk of cervical cancer (by a factor of 1.7 compared to women who attend screening with clinician collection).

^e^ All else equal, the estimates for the proportion of women switching from clinician to self‐collection tended to be lower than the levels reported in this table if screening were to also satisfy the criteria for cost‐effectiveness.

^f^ With the given increase in screening attendance among underscreened, high‐risk women and the given relative sensitivity, a screening programme would remain effective even when 100% of well‐screened women switched to self‐sampling.

This framework lends itself to identification of the core challenges relevant to the implementation of self‐sampling and potential mitigations (Table 3). Now that several high‐income countries are accelerating their plans to introduce self‐sampling as a routine offer not only to nonparticipating women, but to all women of screening age irrespective of their previous engagement with clinician‐based programmes,^17^, ^18^, ^19^, ^20^, ^21^, ^22^ these considerations are time‐critical.

**TABLE 3:** Anticipated risks for the implementation of HPV self‐sampling as a cervical screening offer for all women, and suggestions for mitigation, with the required information to be collected either through research or through on‐going monitoring within real‐world implementation efforts

Anticipated risk	Contributing factors	Suggestions for mitigation
Uptake among underscreened women	Low participation	Identify reasons for not collecting a sample that can be turned into targets for intervention through, for example, innovative qualitative research
		Consider tailored wording and the design of the invitation letters, develop consistent messaging appropriate for the specific target (sub‐)group(s)
		Optimise women's experience with the handling of the sample from collection to posting, and develop accessible platforms to order self‐sampling kits if using an opt‐in strategy
		Identify locally preferred self‐sampling device(s) and determine the feasibility of allowing women to choose a preferred device
		Consider the feasibility of nonmailout self‐sampling kit delivery models, for example, for specific subgroups of the target population
		Signpost where information and other resources are available, and consider outreach initiatives for hard‐to‐reach groups
		Engage with and provide core training to primary care and other health care practitioners involved in cervical screening
Detection of high‐grade CIN	Lower test sensitivity	Identify and optimise sample and workflow parameters causing lower detection of lesions in primary screening
		Identify ways to enhance sample workflow and processing steps to optimise biospecimen quality for robust HPV testing
		Undertake in‐depth comparisons between self‐sampling devices and assay combinations
		Provide improved real‐life data on the detection of high‐grade CIN: Stratification by the women's risk (eg, by age, deprivation, screening history) Longitudinal data to better understand the risk of lesions after negative self‐taken tests Observational data from early adopters
		Standardise workflows between laboratories and, if possible, between screening settings
		De‐prioritise approval and funding of studies undertaken in nonrepresentative populations without histological endpoints, to increase confidence in the technology and accelerate regulatory approval
		Be reactive to new commercial offers of platforms and tests that are deliberately tuned to detect HPV in self‐collected samples
		Provide data on test accuracy and adequacy for urine with similar degrees of robustness as has been made available for vaginal self‐sampling
		Consider implementing a safety net for women with negative HPV tests on self‐collected samples, for example, in the form of shorter routine recall intervals than for women screened with clinician‐collected samples
		Enhance access to quality‐assured biobanked material representative of primary screening populations, with reliable linkage to disease outcomes
	Lower adherence to clinician‐collected triage testing	Identify effective interventions to reduce nonadherence with clinician sampling for cytology
		Prioritise development and validation of triage methods that can be performed on a self‐sample, with fresh or biobanked sample material
		Determine any other parts of the follow‐up process, for example, early recall (for triage‐negative women) or colposcopy referral (for triage‐positive women), that also result in a lower adherence

## 3 UPTAKE IN UNDERSCREENED WOMEN

Many research studies from high‐income countries have shown that self‐collection can engage previously underscreened women in providing an HPV sample. The observed proportions have ranged between ~10% and ~30%.^23^, ^24^, ^25^, ^26^, ^27^, ^28^, ^29^, ^30^, ^31^ With coverage rates for clinician sampling often reaching ~70%,^5^, ^6^ this means that self‐sampling may be able to engage an additional ~3% to 9% of the target population.

Data from early‐adopter programmes show a more varied picture. The programme in the Danish Capital Region, which has contacted women overdue for screening since 2017, reported that 17% of the women returned a self‐collected sample.^32^ This would increase screening coverage from ~73% to ~77%.^32^, ^33^ Some of the women who were offered these self‐collection kits underwent clinician sampling instead, although for them the role of the self‐sampling invitation remains unclear. Elsewhere, the results have been far less encouraging. In Australia, self‐sampling for underscreened women has been available since 2018. From the catchment area of a laboratory in Victoria, where the uptake with clinician sampling was ~60%, only 1067 underscreened women provided self‐collected samples in the first 17 months of the service compared to 290 000 clinician samples analysed during the same period^34^; if this rate was sustained, the overall screening coverage would increase by <0.5%. In the Netherlands, all women invited for cervical screening have been able to order a self‐sampling kit since 2017. Before the disruption caused by the SARS CoV‐2 pandemic, around 8% of participants chose self‐collection.^5^, ^35^ Around 29% of the women who chose self‐collection were previous nonparticipants, compared to 12% among those who chose clinician collection.^36^ Although self‐collection may have motivated more previously unscreened women to provide a sample, the overall screening coverage did not increase.^5^ We discuss the experience from these and other countries in more detail below.

### 3.1 Improving the uptake among underscreened women

The programmes now require in‐depth understanding into why underscreened women do not use self‐collection even when it is offered for free and signposted in an invitation letter. This work may require innovative qualitative studies. Elfström et al, for example, analysed interactions between trial coordinators and the women they approached for a study, and reported that some had been discouraged once they realised that self‐collection did not obviate a clinic visit should they test positive.^27^ Trying to elucidate why some women do not participate in bowel screening, which also relies on self‐collection at home, Kotzur et al interviewed women with different bowel screening histories and categorised them according to their participation in breast and cervical screening.^37^ They compared women who fully followed recommendations for organised screening in Scotland and participated in screening for all three cancers, those who partially followed the recommendations and participated in breast and cervical screening but not bowel screening, and those who did not follow any of the recommendations and remained unscreened for all three cancers. Using our study design, the authors confirmed that women in all three groups expressed disgust at the sample collection process and thus, unlike previous studies, demonstrated that disgust did not discriminate well between participants and nonparticipants. Disgust is, therefore, an imprecise basis from which to develop effective interventions to increase screening participation. The authors then identified a cluster of actionable factors such as perceiving the process as burdensome or forgetting to accommodate sample collection into daily life, which were all more prevalent among nonparticipants. They argued that these could be addressed with pragmatic interventions such as by providing a planning support tool to increase women's ability to complete the test, or stating a deadline in the invitation letter for the sample to be returned to a laboratory.^37^ It is likely that similar research could provide a list of pragmatic interventions to increase the uptake of HPV self‐sampling.

Another area worthy of further investigation is the wording and the format of the invitations. Research studies have tended to include underscreened women, emphasising in their invitation letters that the women were being offered self‐collection because of the higher risk of cancer after having missed previous screening opportunities. When self‐sampling is offered to all women regardless of their screening histories, standard invitation letters may be unable to directly address specific subgroups of women. Furthermore, women have been used to receiving the same format of invitations for clinician sampling for years and may not realise that new‐style invitations offer a wider range of options to collect a screening sample. In the Netherlands, for example, only half of the women who received the standard invitation letter realised that they could now order a self‐sampling kit.^38^ Several programmes still rely on written letters, which is arguably out of step with the digital age. Letters are also static, whereas an online environment may provide real‐time opportunities for the user to seek further clarification and supportive information. Adaptation and customisation of invitations to ensure that they are appropriate for a particular demographic or for subgroups with specific screening histories should be informed through collaboration with experts on the communication of health‐care messages.

Furthermore, programmes may consider a range of options in terms of the design and the distribution of the self‐collection kit. Given the evidence to suggest that women may differ in their preferences for a particular kit,^39^, ^40^ allowing choice of more than one validated option may increase uptake. Another much‐discussed topic has been whether to offer self‐sampling in a mailout delivery model as an ‘opt‐out’ service (whereby a self‐sampling kit is part of an invitation pack sent to all women) or an ‘opt‐in’ service (whereby invited women are asked to order a kit from the screening service; this avoids some plastic waste). Although opt‐out usually results in higher uptake,^27^, ^41^, ^42^ opt‐in can be an effective alternative particularly when supported with suitable communication platforms that simplify the kit ordering process.^28^, ^42^, ^43^ Self‐collection for HPV testing, in fact, allows greater flexibility in approaching underscreened women, and delivery models other than those relying on mailout have also been frequently discussed. In countries with well‐screened populations these have included, for example, an opportunistic offer of a self‐sampling kit in primary care to women attending for unrelated reasons,^44^, ^45^ organising distribution of self‐sampling kits through a network of local pharmacies,^46^ recruitment through home visits by health care workers,^47^ or via community campaigns and other outreach activities.^48^, ^49^ Although such approaches may successfully engage some or even very large proportions of underscreened women to provide a screening sample, they require additional resources. Their suitability for routine use in mass screening programmes would benefit from further study.

The poor uptake of self‐sampling among underscreened women in Australia has been studied thoroughly and attributed to a variety of early‐implementation operational issues, several of which originated from lack of clarity on regulatory approval and on the eligibility criteria.^34^, ^50^, ^51^, ^52^, ^53^, ^54^ Due to insufficient HPV assay manufacturer claims for self‐sampling as ‘intended use’, local validation of a specific self‐sampling utensil was required by the Australian national regulator. This was initially only completed by a single laboratory, likely as a consequence of limited incentives due to a perception of a small target population.^51^ The eligibility criteria used to be relatively restrictive, providing cost reimbursement only in case women were at least 2 years overdue for their screening test.^34^, ^51^, ^53^ Initially, the policy also required that women perform self‐collection in health care facilities rather than at home,^55^ which limited access for women who do not engage with health care.^53^ Further reducing the effectiveness of this implementation was the need to confirm eligibility in real‐time by looking up an individual woman's screening history, hindered by a delayed arrival of certain functionalities of the national screening register.^34^, ^50^, ^51^, ^52^ This was compounded by underinvestment into promoting the availability of HPV self‐sampling as a new feature of the renewed national screening guidelines^51^, ^52^ and insufficient education and training for health care practitioners, among whom a significant proportion expressed lack of confidence and comfort in recommending self‐sampling to eligible women.^52^, ^54^ Although the Australian programme is now addressing these issues,^56^ the learnings are highly instructive for other programmes. Australian experience shows that uncertainty regarding technical specifications of new screening technologies and insufficient investment into its promotion can easily translate into unclear public messaging and, ultimately, into not reaching the target population.^50^, ^51^ In general, navigation of the process for validation and approval of self‐sampling devices in cervical screening is a global issue that we further discuss below.

Finally, women from more deprived backgrounds and those unscreened for a very long time (two prominent risk factors for cervical cancer) may be particularly difficult to engage even with self‐collection.^25^, ^28^, ^32^, ^57^ Reasons include, for example, not having a fixed address, a generalised suspicion of health care interventions, comprehension challenges and coping with issues that mean screening cannot compete for time, resources or attention. To improve screening participation in these groups of women, programmes may need to consider bespoke outreach interventions.^58^, ^59^

## 4 SWITCHING OF WELL‐SCREENED WOMEN TO SELF‐SAMPLING

Observational data from real‐world implementation describing the frequency of substituting clinician with self‐collection have been scarce. Notably, before the SARS CoV‐2 pandemic, self‐sampling was used by only 8% of participants in the Netherlands but in 2020, this proportion increased to 16%.^5^ This proportion is likely to increase further, as the National Health Council recommended in 2021 that a self‐sampling kit should be included in the invitation letter, rather than being available on demand.^20^

Research studies have typically reported that half or more of routinely screened women expressed a preference for self‐sampling over clinician sampling, and that those who tried out self‐sampling are more likely to seek it out again in the future.^60^, ^61^, ^62^, ^63^, ^64^, ^65^, ^66^, ^67^, ^68^ With changes that may tempt women toward self‐sampling such as an increasing variety of self‐sampling options to choose from (including urine sampling discussed below), an increasing propensity for programmes to offer national distribution of self‐sampling kits to entire target populations,^20^, ^69^ and increased familiarity with at‐home microbial testing owing to the SARS CoV‐2 pandemic, self‐collection may eventually become the chosen cervical screening method for millions of women.

Within this context, it may be useful to add that switching from clinician‐collection to self‐collection among a certain proportion of well‐screened women would be a likely outcome even when self‐collection is only offered to underscreened women.^70^ As underscreened women would typically be invited for self‐sampling after a specific period of nonresponse to a screening invitation, well‐screened women wishing to be part of that offer would need to actively delay their participation to qualify. Hence, unauthorised extension of screening intervals among a proportion of previously well‐screened women may be one of the unintended consequences of limiting the self‐sampling offer to women overdue for screening.

When HPV self‐sampling leads to a similar detection of CIN2+ as HPV testing relying on clinician‐collection, screening can remain highly (cost‐)effective even when all well‐screened women switch from clinician‐collection to self‐collection.^51^, ^71^ Consequently, addressing any issues associated with a lower detection of CIN2+ through self‐sampling will be key in avoiding an unintended deterioration in the effectiveness of screening programmes (Tables 1 and 2).

## 5 SENSITIVITY OF SELF‐SAMPLING FOR THE DETECTION OF CIN2+

One of the most influential pieces of work on this topic has been the meta‐analysis in which the relative sensitivity for the detection of CIN2+ with self‐sampling vs clinician sampling was estimated at 0.99 (95% CI: 0.97‐1.02), particularly if sensitive target amplification HPV DNA tests were used.^41^ As others have pointed out, however, these findings are challenging to generalise to populations served by quality‐assured screening programmes because the meta‐analysis included predominantly small studies in populations without access to organised screening.^72^, ^73^ It was also dominated by studies of women referred to colposcopy because of abnormal cytology. CIN2+ associated with abnormal cytology appear to be more easily detectable with any HPV method than CIN2+ associated with negative cytology,^74^ potentially because abnormal cytology tends to be associated with higher HPV viral loads.^75^ The high relative detection of CIN2+ for self‐collection vs clinician samples observed in the meta‐analysis is therefore at risk of spectrum bias^76^ that is, may overestimate the relative detection that would have been observed in primary organised screening where women are managed according to their HPV result rather than cytology. The meta‐analysis did, in fact, include four primary screening studies using target amplification HPV assays. However, three of those were undertaken in previously unscreened populations where the characteristics of the prevalent HPV infections tend to be different than in well‐screened populations, and used assays that have not been used for large‐scale screening implementation in high‐income countries.^77^

Recent reports from settings with long‐standing screening programmes show a more nuanced picture. Randomised studies from Sweden and the Netherlands reported similar detection of high‐grade CIN for self‐sampling vs clinician sampling arms.^72^, ^78^, ^79^ These studies employed HPV assays that have either not been commercialised or clinically validated according to the international metrics.^77^ The uptake was higher in self‐sampling arms, albeit to varying degrees and further stratification of CIN detection by women's screening histories would be informative.

Another Dutch study used data from the early phase of the routine implementation of self‐sampling in the population‐based screening programme.^73^ Our study, whose nonrandomised and unpaired design may be seen as a limitation, reported a relative detection of CIN2+ of 0.91 (95% CI: 0.88‐0.96) for HPV self‐sampling using the cobas 4800 assay on samples collected with the Evalyn brush. The estimate was 0.79 (95% CI: 0.67‐0.92) when only women who participated in the previous screening round were included. A reanalysis of these data, adjusting for screening history, age, screening region and various sociodemographic characteristics including income, household composition and migration background, reported the relative detection of CIN2+ as 0.76 (95% CI: 0.70‐0.82) overall and 0.73 (95% CI: 0.65‐0.83) among previously screened women.^36^ The authors considered the resuspension volume (20 mL) of self‐collected samples in the PreservCyt liquid as a potential contributor to these findings,^73^ which further underscores the need for end‐to‐end optimisation of sample processing protocols (see below). Finally, a paired study in which consecutive women invited by the Scottish organised programme contributed both clinician and self‐collected samples, reported a relative detection of CIN2+ of 0.93 (95% CI: 0.90‐0.98) for samples which were self‐collected with the cobas PCR female swabs and tested on the cobas 4800 assay.^80^

These two real‐world studies suggest that the sensitivity of self‐sampling for the detection of CIN2+ may be almost 10% lower than that achieved with clinician sampling on average, and potentially even lower among regularly screened women (‘switchers’). The reasons for this difference have not yet been fully elucidated. Potentially, weaker signals in regularly screened women who tend to attend their routine recall with more recent HPV infections may account for some of the observed difference; this would, however, require further study.

Recognising the current shortcomings of the testing technologies and supporting their further development is a key task for stakeholders involved in the implementation of HPV self‐sampling. This is underscored by the risk that a lower detection of CIN2+ could manifest itself after several years as a higher incidence of interval cancers,^2^ resulting in loss of life and adding pressure to the already complex task of steering a screening programme.

### 5.1 Improving the sensitivity of self‐sampling for the detection of CIN2+

Most HPV assays and their operational protocols were calibrated with clinician‐collected samples in mind. Self‐collected samples appear to have a lower concentration of viral material (cellularity) than clinician‐collected samples; furthermore, cellularity may differ between self‐sampling devices, and these differences may influence clinical performance.^73^, ^81^, ^82^, ^83^, ^84^ Thus, it seems reasonable to suggest that optimisation on self‐collected samples could enhance HPV testing performance. While re‐calibration of assay cut‐offs to suit self‐collection is not a trivial undertaking, particularly if regulatory approval is sought, it may nevertheless be justified if self‐collection is to be deployed increasingly for routine use.

Another relevant issue is what constitutes a valid vs invalid self‐sample for HPV testing and whether this is differentially affected according to population, device, assay or preanalytical stages including pretest storage time or temperature (eg, while in transport from a woman's home to a laboratory). Optimisation of these technical aspects will benefit from more granular reporting on preanalytical steps and the definitions used for unsuitability and invalidity.^85^, ^86^

Currently, preanalytic processing of self‐collected samples remains relatively manual compared to the systems set up for clinician‐taken samples, to an extent that they may be unsuitable for high‐throughput routine settings.^82^ Due to this and other factors including the lack of longitudinal performance data, HPV self‐sampling technology still lags behind with respect to formal validation and inclusion in manufacturer claims, with only few manufacturers having a CE‐marked claim for self‐collected samples^87^ and, to our knowledge, at time of preparation of this review, no manufacturer has an FDA‐approved claim. Given the increased appetite for HPV self‐sampling, it is very likely that an increasing number of formally approved options will emerge from the commercial sector. Screening programmes and funders may also prioritise primary screening projects that have the potential to facilitate best‐practice around operationalising of HPV testing on self‐collected samples including all preanalytical stages. Shared international learning from project and programme experience will be helpful in the creation of quality assurance frameworks and guidance consistent with good clinical and laboratory practices while remaining mindful of the changing technology.

While waiting until longitudinal evidence becomes available, self‐sampling policy can be guided by the outcomes of well‐designed cross‐sectional validation studies so as not to unduly delay its implementation. In light of the low specificity of HPV infections for the detection of CIN2+, however, studies should ideally continue to validate the performance of self‐sampling tests against histologically confirmed endpoints,^88^, ^89^ even if virological endpoints appear to lead to quicker answers.^90^

This work could be expedited by having access to biobanked collections of samples from primary screening populations that have been linked to subsequent CIN2+ outcomes. Stored samples represent credible biospecimens for HPV testing and have been used successfully to expedite the clinical and technical performance validation of several HPV assays, including through the Meijer noninferiority validation criteria or the VALGENT exercise.^91^, ^92^, ^93^ Furthermore, given the low prevalence of CIN2+ in well‐screened populations, biobanks provide the opportunity to enrich for primary screening samples associated with CIN2+ to support retrospective assessments. Clearly, biobanks require both human and physical resource as well as strong support to ensure appropriate governance is in place and data linkages are robust.^94^ However, having well‐annotated archives that include self‐taken samples provides opportunities to assess the ever‐expanding list of emerging HPV assays, including in terms of comparative performance. In NHS England, as part of a programme‐endorsed technical evaluation of self‐collection devices and tests within clinical and primary care settings, a biobanking component has been included for these reasons.^21^

### 5.2 Implications of low compliance with triage testing on the detection of CIN2+

Women with HPV‐positive self‐collected samples are required to make an appointment for a new, clinician‐collected, sample for cytological evaluation, which is at present the most thoroughly validated method for triage to colposcopy.^2^ If women do not take this step in the screening process, they are most likely unable to proceed to colposcopy and receive a diagnosis and treatment of any prevalent CIN2+ lesions. Because screening is most effective if progressive lesions are treated early, this would mean the benefit they gained from self‐collection was negligible. In the Dutch real‐world implementation, 78% to 79% of HPV‐positive women attended for clinician‐collected triage sampling within a year of the screening sample.^5^ Somewhat higher adherence, ~90%, was observed among underscreened women in routine implementation in Denmark and in a trial using a mailout model of self‐sampling kit distribution in Australia,^32^, ^95^ although in the latter case <60% of women with HPV‐positive, cytology‐negative screening tests adhered with early‐recall testing.^95^ Similarly low adherence with early recall testing has been reported in the Australian national statistics for HPV‐positive women whose samples were taken by a clinician.^96^ In other countries, however, the success of self‐sampling strategies in retaining HPV‐positive women on their pathways to diagnosis will be scrutinised against considerably more effective follow‐up adherence models. In England, for example, the proportion of women who were HPV‐positive on a clinician‐collected sample and completed early recall testing in 12 and 24 months after initially negative cytology was ~85%, whereas the proportions undergoing colposcopy remained at >90% regardless of whether the woman was referred at baseline or after one of the two early recalls.^97^

Working on the assumption that women who do not attend for clinician‐collected triage testing have the same risk of CIN2+ as women who do, nonadherence further reduces the relative detection of CIN2+ with self‐sampling from just over 90% as described above to just over 70%. This is the most favourable estimate from the Dutch programme (91%^73^ × 79%),^5^ although it could be as low as around 60% (73%^36^ × 78%^5^). Even with 90% attendance for triage and 90% relative sensitivity, the detection of CIN2+ would still lag well behind that of clinician collection, at ~80%.

### 5.3 Improving the triage pathway

Interventions improving adherence with clinician sampling when the triage approach obligates it—at baseline and at early recall—will be key in achieving high detection of CIN2+ in women screened with self‐collection. Previous studies have shown that patient‐provider communication using for example, reminders or counselling and educational sessions can improve adherence after abnormal screening tests.^98^ How these interventions could be optimally applied within the context of self‐collection would require further study.

Importantly, molecular biomarkers which can be applied directly on the self‐collected HPV‐positive screening test would bypass the issue of attendance for a clinician‐collected sample. While limited genotyping for HPV16/18 would be practically straightforward and may be routinely available according to the read‐out of the screening test, it requires a second triage test (this is usually cytology) to identify the group of women with CIN2+ lesions caused by other high‐risk genotypes. Increasing the genotyping capacity beyond HPV16/18 may well have promise, and it is interesting that a greater number of new HPV platforms provide assay read‐outs as risk ‘clusters’ where the 14 genotypes traditionally considered high‐risk are aggregated into tiers according to their ultimate risk of high‐grade CIN. Much work has also been devoted to the assessment of methylation biomarkers as a triage option for self‐taken samples and data show a good sensitivity particularly at the level of CIN3+. Various methylation targets (viral, host, both) and protocols have been described.^99^, ^100^, ^101^ However, scalability and automation remain an issue and efforts to improve this are welcome.^102^ Finally, it is worth noting that the expansion in molecular diagnostics in the last 2 years has followed an unprecedented acceleration due to SARS CoV‐2. Collateral value may well be conferred to HPV‐relevant biomarker developments because of increased access to skilled workforce and sophisticated platforms.^103^

### 5.4 Emerging collection methods

Urine is fast becoming another viable option for woman‐friendly collection of cervical screening samples. Studies using a new collection device (Colli‐Pee) have shown increased detection and improved sensitivity for CIN2+ compared to collection of urine in a sterile pot.^104^, ^105^ This device collects first‐void urine, which contains washed away cervical exfoliated cellular debris containing significantly more HPV DNA compared to subsequent urine fractions.^106^ However, studies remain few and the true clinical accuracy needs to be proven in larger primary screening populations studies using a range of HPV PCR assays.

Optimisation of testing protocols is necessary to provide assurance for a role of this sample type in population‐based screening. The crucial considerations include those regarding the choice of the preservative, concentration volumes, the timing for adding the urine to the preservative to prevent DNA degradation resulting in invalid or false‐negative HPV samples, and stability at different temperatures during transport to the laboratory.^107^ Additionally, all considerations regarding triage discussed for vaginal self‐collection also apply to urine self‐collection, although proof‐of‐concept work has demonstrated that urine can be used with limited genotyping and methylation analysis.^108^ This is currently being developed in feasibility studies, which are beginning to provide additional reassurance for the use of methylation analysis to discern between low‐grade and high‐grade histological lesions in HPV‐positive women.^109^

## 6 CLOSING REMARKS

Delaying an implementation of new‐generation life‐saving screening tests costs lives,^110^ especially considering the potential of HPV self‐sampling to accelerate the elimination of cervical cancer globally. HPV self‐sampling has other nontrivial benefits in cervical screening, such as a reduction of the psychological burden which some women experience in association with providing a screening sample.^4^, ^65^, ^68^

Nevertheless, although various modelling studies have by now helpfully demonstrated that HPV self‐sampling could be a (cost‐)effective addition to cervical screening programmes,^14^, ^16^, ^51^, ^71^ an implementation leading to a substantially lower detection of CIN2+ without increasing participation among high‐risk underscreened women could reduce the number of lives that could be saved through screening.^14^, ^16^ In light of this, the observations from early adopters reviewed here have been helpful in identifying various levers which could be used to steer the implementation process and, acknowledging the drawbacks of the currently available technologies, offer HPV self‐sampling to women using models consistent with the principles of informed choice. Precaution and mitigations are particularly relevant for countries with established call recall programmes that have achieved high screening uptake and have traditionally operated a very high‐quality screening service, which places a high burden of proof on new technologies.

Without advocating for the pursuit of the perfect at the expense of the good, translation into real‐world implementation now needs to focus on mitigations that would prevent unfavourable scenarios (Table 3). Planning that considers local contexts will be crucial in supporting a streamlined self‐sampling service. Pilot studies may be helpful in this regard and would also allow screening programmes to fine‐tune any governance and communication issues, test logistics, triangulate communication between the different levels of care, and test external but linked systems such as postal services.

The amount of the work ahead of us remains substantial, but we hope that this review provides a good starting point and are certain that satisfactory solutions could be within sight if we enhance international collaboration between experts from various disciplines.

## AUTHOR CONTRIBUTIONS

**Matejka Rebolj:** Conceptualisation; Literature review; Writing (original draft); Writing (review and editing); Decision to submit. **Alexandra Sargent:** Literature review; Writing (review and editing); Decision to submit. **Sisse Helle Njor:** Literature review; Writing (review and editing); Decision to submit. **Kate Cuschieri:** Literature review; Writing (review and editing); Decision to submit. The work reported in the article has been performed by the authors, unless clearly specified in the text.

## CONFLICT OF INTEREST

Matejka Rebolj: Public Health England provided financing for the epidemiological evaluation of the English HPV screening pilot and the HPValidate study in HPV self‐sampling; member of various expert groups providing advice to the English Cervical Screening Programme including on HPV self‐sampling; attended meetings with various HPV assay manufacturers; fee for lecture from Hologic paid to employer. Alexandra Sargent: Member of various expert groups providing advice to the English Cervical Screening Programme including on HPV self‐sampling; holds an honorary contract with The University of Manchester to support research into HPV testing in urine samples and Professional Clinical Advisor to the English Cervical Screening Programme. Sisse Helle Njor: Declares no conflict of interest related to this review. Kate Cuschieri: Research funding or consumables to support research in the last 3 years from Cepheid, Euroimmun, GeneFirst, SelfScreen, Hiantis Seegene, Roche, Abbott and Hologic, paid to employer. Professional Clinical Advisor to the English Cervical Screening Programme; member of various expert groups providing advice to the English Cervical Screening Programme including on HPV self‐sampling; member of the working group that has provided advice to the Scottish Cervical Screening Programme on the potential use of HPV self‐sampling within the programme.

## ETHICS STATEMENT

No ethics approval has been sought as this review was based on previously published data.

## Data Availability

The authors cannot provide access to the data discussed in the article, as the review was based entirely on previously published data collected by multiple research groups.
